# Automatic On-Line Purge-and-Trap Sequential Injection Analysis for Trace Ammonium Determination in Untreated Estuarine and Seawater Samples

**DOI:** 10.3390/molecules25071569

**Published:** 2020-03-29

**Authors:** Athina Dimitriadou, Aristidis Anthemidis

**Affiliations:** Laboratory of Analytical Chemistry, Department of Chemistry, Aristotle University, 54124 Thessaloniki, Greece; athina.dimitriadou@gmail.com

**Keywords:** automation, purge-and-trap, sequential injection analysis, fluorimetric, ammonium determination, environmental samples

## Abstract

An innovative automatic purge-and-trap (P&T) system coupled with fluorimetric sequential injection (SI), for the on-line separation and preconcentration of volatile compounds, is presented. The truth of concept is demonstrated for the ammonium fluorimetric determination in environmental water samples with complex matrices without any pretreatment. The P&T flow system comprises a thermostated purge-vessel where ammonium is converted into gaseous ammonia and a trap-vessel for ammonia collection. This configuration results in matrix removal as well as analyte preconcentration, avoiding membrane-associated problems. All the main parameters affecting the efficiency of a P&T system were studied and optimized. The proposed method is characterized by a working range of 2.7–150.0 μg L^−1^ of NH_4_^+^, with a detection and quantification limit of 0.80 and 2.66 μg L^−1^, respectively, for a 10-mL sample consumption. The accuracy of the method was assessed by recovery assays in seawater, estuarine, and lake water samples as well as by the analysis of standard reference material.

## 1. Introduction

Automatic on-line analytical methods based on flow injection (FIA) and sequential injection (SIA) analysis have been proved to be versatile, fast, accurate, robust, and efficient analytical tools [1]. These methods have led to the development of less invasive chemical assays with low reagent consumption and waste generation, fulfilling the principles of Green Analytical Chemistry (GAC) [2]. A remarkable advantage of automatic on-line systems is their ability to integrate and handle a plethora of sample pretreatment techniques, including sample separation and preconcentration [3,4].

Ammonium is an analyte of high environmental interest due to its double role as a significant ecological nutrient in the nitrogen cycle of seawater and a common pollutant in alkaline aquatic ecosystems. In natural waters, the ratio of ammonia to ammonium is significantly dependent on pH level and temperature. At alkaline environments, toxic ammonia is the predominant form, which is an indicator of water quality. Various flow-based methods have been implemented for ammonium determination in water samples typically based on the spectrophotometric indophenol blue or fluorimetric o-phthaldialdehyde (OPA) techniques [5]. In particular, the OPA reaction was modified by replacing mercaptoethanol with sulfite, in order to avoid the interference from organic amine compounds resulting in higher sensitivity and selectivity [6]. Afterwards, the method was further modified for the determination of ammonium in seawater [7]. Regarding the direct analysis of ammonium in natural and seawater samples, it suffers from several interferences arising from complicated matrices and possible dissolved materials [8]. Hence, separation and preconcentration techniques are required to eliminate matrix problems and to enhance the sensitivity and selectivity of the method.

Several approaches for the on-line ammonia gas–liquid separation have been reported and are classified as: a) membrane-based, such as gas-diffusion (GD) [9,10,11] and pervaporation [12,13], b) membranelles-gas-diffusion (MGD) [14,15,16], and c) headspace single drop (HS-SD)-based techniques [17,18]. Membrane-based methods make use of a hydrophobic membrane that allows the diffuse of gaseous ammonia through the membrane, from a donor to an acceptor liquid stream. Nevertheless, serious problems, such as clogging, cracking, and the deterioration of membrane in the GD units, are often observed due to the contact of the membrane with the sample solution. On the other hand, pervaporation units involve an air gap between the membrane and the donor stream in order to overcome the GD limitations. However, reduced diffusion effectiveness has been recorded due to water condensation on the surface of membrane, in pervaporation units [13,19]. Regarding both MGD and HS-SD techniques, they require careful and accurate handling of solution, as well as complicated equipment. Lately, a membranelles automated on-line dual-headspace lab-in-syringe (PA-D-HS-LIS) system for the gas separation of volatile compounds was presented for ammonia determination [20]. This system is based on decreased pressure under increased temperature into the headspace of sample solution for effective ammonia gas release. Then, the generated ammonia is transported to the second headspace of the extraction solution for its dissolution under increased pressure.

Purge-and-trap pretreatment is a well-established methodology for separation of volatile organic compounds (VOCs) in aqueous samples with complex matrices. In principle, an inert gas passes through the water to purge the volatile compounds, from the aqueous to gas phase, which are subsequently collected in an appropriate trap for further analysis [21]. Compared to GD and MGD, the P&T configuration can process higher sample volumes in the pretreatment steps, thus enhanced sensitivity can be achieved [22]. As far as we know, only a few P&T methods for ammonium determination in a manual operation, have been presented [23,24].

In this study, a novel automatic dynamic purge-and-trap platform based on sequential injection analysis (P&T-SIA) for the separation and preconcentration of volatile compounds, has been developed for the first time. The proposed system eliminates the serious problems arising from the use of membrane in the GD and pervaporation units. The effectiveness of the P&T-SIA system was demonstrated for ammonium determination. Gaseous ammonia is generated by adding an appropriate volume of NaOH solution to the sample. After its transportation, ammonia is dissolved in the acidic trapping solution and is determined by fluorimetric sequential injection analysis (SIA). All the main factors influencing the efficiency of the P&T system, such as purging flow rate, purging time, temperature, liquid depth, and the concentration of the trapping solution, have been commented and optimized. The applicability of the P&T-SIA system has been evaluated in seawater, estuarine, and lake water samples for ammonium determination.

## 2. Results and Discussion

### 2.1. Optimization of the Automatic Purge-and-Trap System

The aim of the present study is to develop and evaluate an effective automatic on-line purge-and-trap system, which is able to be connected with the miniSIA device for the fluorimetric determination of ammonium or other volatile compounds. For setting up the P&T-SIA system, several key issues should be considered. Some of them include the pH, the temperature, and the volume of the sample solution, the flow rate of the purge gas, the time of purging, as well as the type and the volume of trapping solution.

#### 2.1.1. Sample Alkalization

In aqueous solutions, ammonia exists simultaneously as two forms: the free unionized gaseous ammonia (NH_3_) and as the ionized ammonium ion (NH_4_^+^). Since the p*K*_a_ of ammonium ions is 9.26, the pH value of the sample should be higher than that value to ensure the formation of ammonia is favored. The chemical equilibrium in the water of the two nitrogenous forms (ammonia and ammonium ion) reveals that a 100% conversion of NH_4_^+^ to NH_3_(g) requires a pH value higher than 11.5 at 20 °C. However, higher values than pH > 13 are recommended for trace ammonia removal from water due to its high solubility [23,24].

Gaseous NH_3_ is produced into the purge vessel from the ammonium ion NH_4_^+^ by sample alkalization with NaOH. Thus, for 10.0 mL of sample volume, a volume of 1000 μL of 1.0 mol L^−1^ NaOH aqueous solution was employed for ensuring a pH higher than 13 in the sample solution.

#### 2.1.2. Trapping Solution

Ammonium gas dissolution is higher in slightly acidic solution than in double-de-ionized water (DDW), as it is shown from preliminary experiments. On the other hand, when ammonium concentration is at mg L^−1^ levels, higher acid concentration is necessary for the best trapping efficiency [25]. Thus, considering the very low concentrations of ammonium (ppb levels) in the samples, the versatility and the requirements of an miniSIA fluorimetric detection system, a 0.001-mol L^−1^ hydrochloric acid solution was adopted as the trapping solution. It should be mentioned that low volumes of the trapping solution result in a high preconcentration ratio. Thus, for higher sensitivity, a volume of 300 μL of HCl solution into the trap-vessel was used throughout the study.

#### 2.1.3. Temperature Effect

Increasing the temperature of the sample solution inside the purge-vessel (PV), causes the decrease of ammonia solubility, as arises from van’t Hoff’s equation [26]. Raising the temperature increases the kinetic energy of the molecules. Therefore, a higher kinetic energy causes more actuation between the molecules that break the intermolecular bonds and escape from the solution, increasing the generation of the gaseous ammonia [20]. The proposed purge-vessel involves a lab-made heating device to adjust the temperature of the sample solution during purging. The effect of the temperature on the fluorescence intensity has been studied in the range 20–80 °C and the obtained results are presented in Figure S1 (Supplementary Material). Temperatures above 80 °C were not applied in order to avoid the possible occurrence of boiling phenomena in the purge vessel and the production of water vapor, which then can be condensate in the tubing and in the trapping vessel. As the recorded signal increased throughout the studied range, for high sensitivity, 80 °C was adopted for further experiments.

#### 2.1.4. Sample Volume

In principle, purging efficiency is affected by several factors, such as Henry’s law constant, temperature, the flow rate of the purge gas, purging time, as well as the number and the size of the purge gas bubbles. Another significant parameter is the depth of the solution in the purge vessel, which is proportional to the sample volume. If all other parameters are constant, a higher solution depth results in more bubbles in the liquid phase, increasing the interfacial area and the remaining time of the bubbles in the solution.

**Figure 1:**
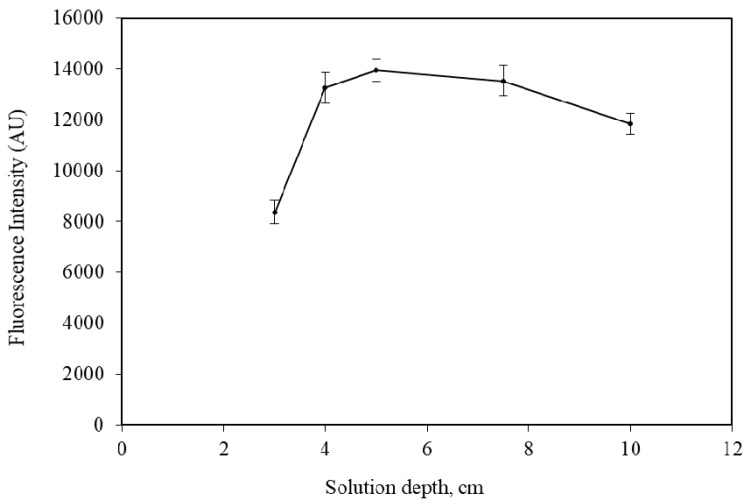
Effect of solution depth in purge-vessel on the intensity of 5.0 μg NH_4_^+^. The error bars were calculated based on standard deviation (± 1 s). NaOH volume = 1000 μL (1.0 mol L^−1^ NaOH); Trapping solution = 300 μL, 0.001 mol L^−1^ HCl; Purge-gas flow rate = 75 mL min^−1^.

In order to estimate the effect of the solution depth on the purging efficiency, five purge-vessels with the same internal diameter (i.d. = 16 mm) but at various lengths, were tested. Specifically, for a solution depth between 3.0 and 10.0 cm, an appropriate sample volume was added into the PV, which ranged from 6.0 to 20.0 mL, respectively. Each vessel contained a fixed amount of 5.0 μg NH_4_^+^. The obtained results are presented in Figure 1, showing that short depth resulted in lower efficiency, as is expected. The optimum depths vary between 4.0 and 7.0 cm. For higher depths, the effectiveness is decreased to some extent, as it is also referred previously [27].

#### 2.1.5. Effect of Purge-Gas Flow Rate and Purging Time

The effect of the flow rate of the purge-gas on the sensitivity of the method was examined in the range 10–100 mL/min and the recorded results presented diagrammatically in Figure S2 (Supplementary Material). As is shown, the intensity increased by increasing the flow rate of argon gas and leveled off for values higher than 75 mL/min. At flow rates higher than 90 mL/min, there was a possible transportation of small drops of the liquid through the tubing. Hence, the optimum flow rate of argon gas was set at 75 mL/min.

Typically, flow analysis methods are not based on thermodynamic equilibrium for their results. In this concept, the purging and trapping operation is not necessary to be completed at the time of measuring. Moreover, the time of purging positively affects the sensitivity of the method and can be considered as preconcentration time. The effect of purging time on sensitivity is shown in Figure S3 (Supplementary Material). It was examined from 1 to 15 min and it was found that the intensity increased by increasing the purging time, while it was leveled off after 10 min. Consequently, 10 min was adopted as the optimum purging time.

### 2.2. Interferences

An inherent feature of the purge-and-trap system is the effective separation of volatile compounds, such as ammonia from the matrix of the sample, eliminating the interferences.

Possible gases, such as CO_2_, SO_2_, and H_2_S are transformed into non-volatile ions CO_3_^2−^, SO_3_^2−^ and HS^–^ by alkalization with NaOH. Hence, they remain in the PV and do not interfere. The developed method is possibly affected by metal ions and volatile amines, such as primary amines. The interference studies were performed using a 50.0-μg L^−1^ NH_4_^+^ standard solution, and a relative deviation of less than 5% was considered as interference. Commonly encountered matrix components in natural water, such as metal ions K^+^, Na^+^, Ca^2+^, and Mg^2+^, were examined individually for each interferent and found to be tolerated at least up to 500 mg L^−1^, while NaCl was tolerated up to 35 g L^−1^. Other metals that could act as interferences are Zn^2+^, Fe^3+^, Al^3+^, Cu^2+^, and Ag^+^, which is used as a disinfectant in water recycling systems. No interference was observed for these metals at concentration levels up to 1.0 mg L^−1^. Primary amine (CH_3_NH_2_) can be tolerated at least up to 0.2 mg L^−1^.

### 2.3. Figures of Merit

The figures of merit of the proposed method (P&T-SIA) under the optimum conditions for ammonium determination were calculated as follows. Under the optimum conditions, for a sample volume of 10.0 mL, the working linear range was between 2.7 and 150.0 μg L^−1^ of NH_4_^+^ with a correlation coefficient (r^2^) of 0.9978. The calibration curve was characterized by the following regression equation: intensity = (258.7 ± 13.8) × [NH_4_^+^] + (47.9 ± 986.4), (7-standards; *n* = 5; [NH_4_^+^] expressed in μg L^−1^). The detection and quantification limits were calculated based on the 3 and 10*s* criterion [28] and found to be 0.80 and 2.66 μg L^−1^, respectively. The precision of the proposed method expressed as relative standard deviation (RSD%, *n* = 10), was calculated at 5.0, 20.0, and 100.0 μg L ammonium concentration levels and was found to be 4.8%, 4.2%, and 3.7% respectively. A complete analytical cycle lasts for 900s, resulting in a sampling frequency (*f*) of 4 h^−1^.

For comparative purposes, the performance characteristics of the proposed method and other selected on–line separation methods based on gas diffusion, pervaporation, membranelles-GD, and HS-SD devises, reported in the literature for ammonium determination, are compiled in Table 1. As it is shown herein, the P&T-SIA method reveals satisfactory sensitivity with better detection limits than earlier works [10,12,15,16,17,29,30,31] for ammonium assays. In addition, it should be considered that the proposed system overcomes membrane-related problems and allows for the determination of untreated samples, minimizing the cost of analysis. According to the GAC requirements, the consumption of o-phthaldialdehyde and sodium sulfite is low enough (≤ 40 μL) and generated organic waste is limited. Regarding the time of analysis of the proposed methodology is rather higher than the other methods, even though it can be reduced to some extent by a strict synchronization of the analytical steps.

The accuracy of the presented method was estimated using a standard reference material SRM from NIST CertiPUR^®^ NH_4_Cl in H₂O 1000 ± 10 mg L^−1^ NH₄^+^. After appropriate dilution, the analytical result for ammonium concentration was 935 ± 42 mg L^−1^ (standard deviation, *n* = 3) with a recovery of 93.5%.

The trueness of the P&T-SIA method was also investigated by analyzing two potable artificial water (PAW) and two hygiene artificial water (HAW) samples, and the obtained results were compared with that obtained by the certified method [32]. The recorded results and the *t*_exp_, values are given in Table 2. The overall relative errors were 2.1% and 3.3% for PAW and HAW samples, respectively, indicating the efficient applicability of the developed method in similar water samples. Moreover, since all *t*_exp_ values are lower than the *t*_crit_, 95% = 4.30, no statistically significant differences were found between the two studied methods.

### 2.4. Applications in Spiked Environmental Water Samples

The proposed method has been applied to the analysis of collected (March 2019) environmental water samples namely estuarine, lake, and seawater. The recovery (*R*) values were estimated by analyzing the spiked samples with 20.0 μg L^−1^ NH_4_^+^ concentration level. The recorded results are presented in Table 3. The recoveries varied within the range 94.0%–102.0%, showing the applicability of the developed method for ammonium determination in natural water samples, even in samples with high salinity such as seawaters.

## 3. Materials and Methods

### 3.1. The Automatic Purge-and-Trap Platform

**Figure 2:**
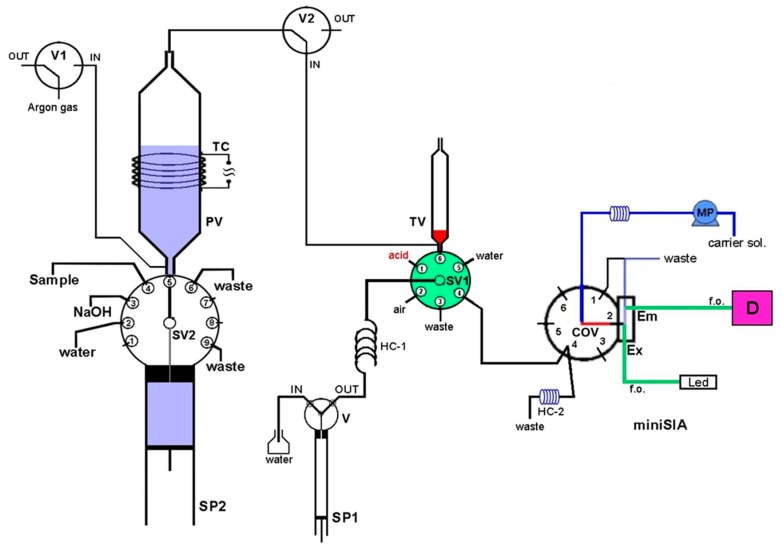
Schematic diagram of the P&T-SIA manifold for ammonium determination. PV, purge vessel; TV, trap vessel; SV1 and SV2, selection valves; COV, chem-on-valve unit with the flow-through cell; V, V1, V2, valves; SP1, SP2, syringe pumps; TC, thermostated coil; HC-1, HC-2, holding coils; MP, milliGUT pump; f.o., fiber optics; D, detector.

**Figure 3:**
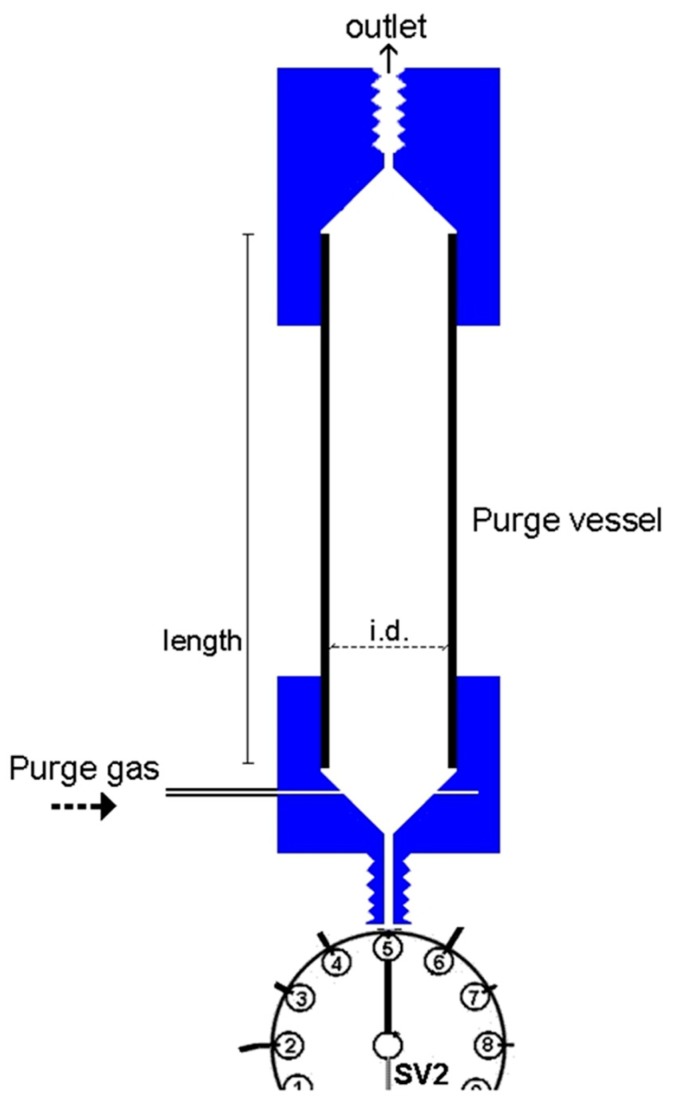
Schematic diagram of the purge-vessel. Internal diameter, i.d. = 1.6 mm; length = 100 mm.

The developed purge-and-trap (P&T-SIA) system for ammonium separation/preconcentration and determination is presented schematically in Figure 2. It consists of a high precision bi-directional micro-syringe pump, SP2 (MicroCSP-3000, FIAlab Instruments, Bellevue, WA, USA), which is equipped with a 5000-μL glass barrel and a nine-position Teflon/Kel-F SV2 selection valve, directly connected at the top of the barrel. The purge-vessel (PV) is made of a borosilicate glass tubing (1.6 mm i.d., 100 mm length) with two push-fit poly tetra fluoro ethylene (PTFE) connectors at its two ends, as is shown in Figure 3. The lower connector is a male flangeless fitting end in order to be connected at the port-5 of SV2 (Figure 1). This connector enables the insertion of the purge gas at the bottom of the PV by four tiny holes, which are connected with a purge gas tubing. The PV is placed in a vertical position in the P&T manifold.

In order to facilitate the gaseous ammonia release, the PV is heated at the appropriate temperature by a lab-made heating device, which consists of a thermostat and a chromium-nickel resistor coil (TC), electrically powered with a 12V/5A power supply. The trap-vessel (TV) is made of a plastic syringe (4.6 mm i.d., 70 mm length), located in the P&T manifold in a vertical position, by using a push-fit connector with a male fitting end. The TV is connected at the SV1 (port-6) of the FIAlab-3000^®^ SI system.

### 3.2. Apparatus

A FIAlab^®^-3000 sequential injection (SI) system (Alitea FIAlab, Bellevue, WA, USA) was used for the handling of the P&T system. This system is equipped with a syringe pump, SP1 (Cavro, Sunnyvale, CA, USA) with a glass syringe barrel of 1000 μL capacity and a two-position (IN/OUT) valve (V), a six-port selection valve (SV1) and a holding coil (HC-1). Two additional three-port, two-position valves, V1 and V2 controlled by the FIAlab^®^-3000 SI system, were used for the purge gas handling. The Micro CSP-3000 syringe pump is interfaced with the FIAlab^®^-3000 system and controlled by a personal computer that runs the FIAlab software for windows v. 5.9.245 (http://www.flowinjection.com).

A miniSIA flow analyzer (https://www.globalfia.com) equipped with an acrylic Chem-on-Valve™ (COV) monolithic manifold was employed for ammonium SI fluorimetric determination. The miniSIA flow analyzer is equipped with a fluorescence flow cell which is located directly in the COV at position-2 (Figure 2). Two fiber optic cables (f.o.) are used for light emission (Em) and excitation (Ex). For the fluorometric detection of ammonium, an Ocean-Optics USB-4000 fluorescence spectrometer with a monochromator set at 425 nm emission wavelength, is used. In addition, a monochrome white LED is used as an excitation light source and has been set at 365 nm excitation wavelength. The recorded fluorescence intensity is given as intensity arbitrary units (AU). More details of the miniSIA flow analyzer are presented in previous work [33]. The quantification of ammonium in miniSIA flow analyzer is based on the OPA reaction, where the ammonia reacts with o-phthaldialdehyde in the presence of a strong reducing agent, sulfite, in order to produce the fluorescent isoindole derivative. The reaction is time and temperature depended.

For the control of miniSIA system and the data acquisition, a laptop running the FloZF 5.2 software (https://www.globalfia.com), is used. The integrated P&T-SIA system is controlled by the two discrete software programs namely FIAlab and FloZF, which are synchronized and activated simultaneously.

### 3.3. Chemicals and Samples

Analytical-grade chemicals were provided by Merck (Darmstadt, Germany, http://www.merck.de) and were used throughout the study. A Milli-Q system (Millipore, Bedford, MA, USA, http://www.millipore.com) was employed for ultra-pure quality water. All ammonium working standard solutions were prepared daily by the appropriate dilution of 5000 mg L^−1^ of NH_4_^+^ stock standard solution with water. A phosphate buffer at 0.1 mol L^−1^ concentration level was prepared by dissolving 13.4 g Na_2_HPO_4_ in 400 mL of water, adjusting the pH to 11.0 with 2.0 mol L^−1^ NaOH solution and diluting into a 500-mL volumetric flask. A solution of 6.0 mmol L^−1^ sodium sulfite was prepared by dissolving 75.6 mg of Na_2_SO_3_(s) in phosphate buffer and diluting to 100 mL. A solution of 15.0 mmol L^−1^ o-phthaldialdehyde (OPA) was prepared by dissolving 201.0 mg of solid C_8_H_6_O_2_ in 20.0 mL methanol and diluting to 100.0 mL with water. Argon (99.997%, Grade 4.7) was used as a purge gas for triggering the release of the ammonia gas from the liquid mixture and transportation to the trap vessel. For ammonium determination by the certified method [32], the following solutions have been prepared properly: a 5% *m*/*v* sodium hypochlorite solution (NaClO); a 11.1% (*v*/*v*) phenol (C_6_H_6_O) solution in ethanol; a 20% (*m*/*v*) alkaline citrate solution; a 0.5% (*m*/*v*) sodium nitroprusside (Na_2_Fe(CN)_5_NO) aqueous solution; and a 1% (*m*/*v*) NaClO in 16% *m*/*v* citrates solution.

For the accuracy validation of the developed method, an ammonium standard reference solution traceable to SRM from the NIST (National Institute of Standard and Technology, Gaithersburg, MD, USA) NH_4_Cl in H₂O 1000 mg L^−1^ NH₄^+^ CertiPUR^®^ has been analyzed, after dilution. Two potable artificial water samples (PAW) and two hygiene artificial water samples (HAW), at 25.0/50.0 μg L^−1^ and at 50.0/100.0 μg L^−1^ concentration levels of NH_4_^+^, respectively, were prepared according to the chemical composition of the recycled water in the International Space Station (ISS). The chemical constitution of them is given elsewhere [33].

The environmental water samples were collected from sampling sites that are located in northern Greece, during March 2019. The estuarine water was from the Strymon river, the lake water was from lake Prespa, and the seawater was from the Thermaikos gulf (Thessaloniki) and the Toroneos gulf (Chalkidiki). The collected samples were filtered through 0.45-μm membrane filter, acidified to ca. pH 2.0 with dilute nitric acid, and stored at 4 °C in acid-cleaned polyethylene bottles prior to analysis. The laboratory glassware was rinsed with water after decontaminated overnight in a 10% (*v*/*v*) nitric acid solution.

### 3.4. On-Line Analytical Procedure

The schematic diagram of the on-line P&T-SIA system for the fluorimetric determination of ammonium is given in Figure 2, while the operational sequence of the proposed method is summarized in Table 4.

At the start-up of the system, the argon purge gas flows through the PV and TV (V1 and V2 in IN position), while the thermostated heater is activated, in order to remove possible ammonia gas molecules from the two vessels. In steps 1-2, 300 μL of trapping solution (0.001 mol L^−1^ HCl) is delivered into the trap vessel, while V1 and V2 are in the “OUT” and “IN” position, respectively. Next (steps 4–7), appropriate volumes of 1.0-mol L^−1^ NaOH and sample/standard solutions are sequentially loaded into the PV by the operation of SP2 through ports 3 and 4 of SV2, respectively. The presence of NaOH creates an alkaline environment inside PV which results in the conversion of ammonium ion to ammonia (gas). Then, the operation of Purge-and-Trap starts (step 10). Specifically, the argon purge gas is flowing in the PV through the sample solution in the PV by switching the V1 in the “IN” position, for a purging time of 600 s. During this step, the sample solution is heated into PV at 80 °C. Consequently, ammonia-gas is released and transported by the argon gas into the TV in order to be dissoluted in the HCl solution (trapping solution).

In the following steps (11–13), the trapping solution is delivered and “parked” in the holding coil HC-2, by the operation of SP1. Then, a small segment of 40 μL is aspirated through port 4 of the COV, into miniSIA analyzer for fluorimetric quantification. The determination of ammonia is based on its reaction with o-phthaldialdehyde (OPA) and sodium sulfite, resulting in the formation of the isoindole derivative. The reaction is depended from the time and temperature. The operational protocol of the miniSIA system, the optimization procedure, as well as all performance characteristics have been presented in detail in our previous work [33]. In the last steps (14–29), a thorough cleaning of the system, including the purge-and-trap vessels and the entire tubing, is performed using ultra-pure water to eliminate any possible carryover phenomenon. Five replicate measurements have been made in all instances.

## 4. Conclusions

An automatic thermostated on-line purge-and-trap system based on the instrumentation of sequential injection analysis was developed for volatile compounds for the first time. The efficiency of the proposed P&T-SIA system coupled to a miniSIA flow analyzer has been successfully demonstrated for direct ammonium separation, preconcentration, and determination in environmental water samples. The presented flow method combines the advantages of membraneless purge-and-trap pretreatment and sequential injection systems. The simplicity, versatility, ease, and low costs of the operation are some distinct advantages that the P&T-SIA flow manifold exhibits. According to the obtained results for ammonium determination, it can be concluded that the newly developed method offers remarkable analytical features, such as good accuracy and reproducibility as well as high sensitivity. The applicability was examined in environmental water samples and specifically in seawater samples. The fluorimetric detection method is regarded as a green and environmentally friendly approach, minimizing the use of toxic reagents in μL levels and generating limited wastes. All these characteristics make the proposed P&T-SIA platform an attractive new tool for determining trace amounts of volatile compounds, suitable for monitoring water pollution.

## Figures and Tables

**Table 1:** Comparative figures of merit of the proposed purge-and-trap platform based on sequential injection analysis (P&T-SIA) method and other on–line separation methods reported in the literature for ammonium determination.

Flow System	Separation Technique	Detection System	Linear Range (mg L^−1^)	LOD (μg L^−1^)	RSD (%)	*F* (h^−1^)	Sample Type	Ref.
MPFS	GD	Chemiluminescence	0.3–0.5	20	< 1.2	50	Tap, river, wastewater	[29]
MCFIA	GD	Spectrophotometry/BTB	0.050–1.0	27	< 1.5	20	Surface and tap water	[30]
SIA	GD	Spectrophotometry/BTB	0.10–1.0	27	< 2	20	Estuarine, river, well, marine water	[10]
FIA	PV	Spectrophotometry/CR - TB	0.2–20	100	< 3	11	Industrial effluents	[12]
FIA	PV	Spectrophotometry/BTB	0.05–50	30	1.9	10/8	Surface, urban sewage, industrial effluents	[31]
LIS	HS-SDME	Spectrophotometry/BTB	up to 0.425	30	< 8	17	River, coastal seawater	[17]
MSFIA	MGD	Spectrophotometry/BTB	10.0–50.0	2200	4.8	11	River, wastewater	[15]
SIA	MBL-VP	Conductivity	0.09–1.44	36	2.0	12	Canal water	[16]
LIS-SIA	PA-D-HS	Fluorimetry	0.15–10.0 *	0.05	3.6	8	Seawater, river, lake, ditch water	[20]
FIA	P&T (off-line)	Fluorimetry	0.18–7.2 *	0.13	4.4	4	Seawater	[24]
SIA	P&T	Fluorimetry	2.66–150 *	0.80	4.2	4	Estuarine, lake, seawater	**

* expressed as μg L^−1^; ** present work; HS-SDME, headspace-single-drop microextraction; MBL-VP, membranelles-vaporization; MCFIA, multi-commuted FIA; MGD, membranelles gas-diffusion; MPFS, multi-pumping flow system; MSFIA, multisyringe FIA; PA-D-HS, pressure-assisted-dual-headspace; P&T, purge-and-trap; PV, pervaporation; BTB, bromothymol blue; CR, cresol red; TB, thymol blue; *f*, sampling frequency; LOD, limit of detection.

**Table 2:** Analytical results obtained by the P&T-SIA method and by the certified method for the determination of ammonium in potable (PAW) and hygiene (HAW) artificial water samples.

Sample	True Value (μg L^−1^)	Certified Method * (μg L^−1^)	P&T-SIA Method * (μg L^−1^)	Relative Error (%)	*t*_exp_
PWA-1	25.0	24.2 ± 1.8	23.8 ± 1.5	1.7	0.831
PWA-2	50.0	48.0 ± 3.2	46.8 ± 2.5	2.5	0.462
Overall relative error				2.1	
HWA-1	50.0	51.6 ± 4.3	48.8 ± 2.4	5.4	2.021
HWA-2	100.0	97.8 ± 8.6	96.6 ± 5.2	1.2	0.400
Overall relative error				3.3	

* Mean value ± standard deviation (*n* = 3).

**Table 3:** Application of the proposed P&T-SIA method for ammonium determination in spiked natural water samples.

Sample Type	Added * (μg L^−1^)	Found * (μg L^−1^)	*R* (%)
Strymon estuarine water		N.D.	
	20.0	19.2 ± 0.9	96.0
	50.0	48.8 ± 1.5	97.6
Prespa lake water		35.2 ± 2.2	
	20.0	54.7 ± 2.7	97.5
	50.0	86.2 ± 2.8	102.0
Thermaikos gulf seawater		12.5 ± 0.5	
	20.0	31.3 ± 1.8	94.0
	50.0	60.2 ± 2.8	95.4
Toroneos gulf seawater		N.D.	
	20.0	20.3 ± 1.3	101.5
	50.0	47.3 ± 2.8	94.6

* Mean value ± standard deviation; N.D, not detected.

**Table 4:** Operational sequences of the P&T-SIA system for ammonia preconcentration and determination.

Step	Valve Position					Operation		Volume (μL)	Flow Rate (μL s^−1^)	Commentary	
	V1	V2	SV1	SV2	V	SP1	SP2				
*Thermostated Heater: ON*										PV is thermostated at 80 °C	
1	OUT	IN	2	5	OUT	Aspirate	-	10	5	Trapping solution into TV	
2	OUT	IN	1	5	OUT	Aspirate	-	300	50		
3	OUT	IN	6	5	OUT	Dispense	-	310	50		
4	OUT	IN	6	3	OUT	-	Aspirate	1000	100	Transportation of NaOH into PV	
5	OUT	IN	6	5	OUT	-	Dispense	1000	100		
6	OUT	IN	6	4	OUT	-	Aspirate	5000	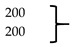	× 2-repeats	Loading of Sample into PV
7	OUT	IN	6	5	OUT	-	Dispense	5000			
10	IN	IN	6	5	OUT	-	-	-	-	Start of Purge-and-Trap operation	
*Purging Time: 600s*										Purge Gas in PV, Transportation and Dilution of NH_3_(g) Separation/Preconcentration	
11	OUT	OUT	2	5	OUT	Aspirate	-	10	5	Delivery of trapping solution into HC-2	
12	OUT	OUT	6	5	OUT	Aspirate	-	400	50		
13	OUT	OUT	4	5	OUT	Dispense	-	410	40		
*Start miniSIA operation*										Measurement/Quantification	
14	OUT	OUT	4	5	OUT	-	Aspirate	5000	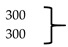	× 2-repeats	Cleaning of PV
15	OUT	OUT	4	6	OUT	-	Dispense	5000			
18	OUT	OUT	4	5	OUT	-	Aspirate	1000	300		
19	OUT	OUT	4	6	OUT	-	Dispense	1000	300		
20	OUT	OUT	4	2	OUT	-	Aspirate	5000	300		
21	OUT	OUT	4	5	OUT	-	Dispense	5000	300		
22	OUT	OUT	4	5	OUT	-	Aspirate	5000	200		
23	OUT	OUT	4	6	OUT	-	Dispense	5000	200		
24	OUT	OUT	3	6	IN	Aspirate	-	500	100		Cleaning of TV
25	OUT	OUT	6	5	OUT	Dispense	-	500	100		
26	OUT	OUT	6	5	OUT	Aspirate	-	500	100		
27	OUT	OUT	3	5	OUT	Dispense	-	500	100		
28	OUT	IN	3	6	IN	Aspirate	-	1000	100		Cleaning of HC-2 of miniSIA
29	OUT	IN	4	6	OUT	Dispense	-	1000	100		

## Supplementary Materials

The following are available online: Figure S1: Effect of the temperature on the fluorescence intensity of 50.0 μg L^−1^ NH_4_^+^. Error bars were calculated based on standard deviation (± 1s). Sample volume = 10.0 mL; NaOH volume = 1000 μL of 1.0 mol L^−1^ NaOH; Trapping solution: 300 μL, 0.001 mol L^−1^ HCl; Purge-gas flow rate = 75 mL min^−1^, Figure S2: Effect of purge-gas flow rate of the on the sensitivity of the method. Error bars were calculated based on standard deviation (± 1*s*). Other parameters as in Figure S1, Figure S3: Effect of purging time on the fluorescence intensity of 50.0 μg L^−1^ NH_4_^+^. Error bars were calculated based on standard deviation (± 1*s*). All other parameters as in Figure S1.

## References

[1] Clavijo S., Avivar J., Suárez R., Cerdà V. (2015). Analytical strategies for coupling separation and flow-injection techniques. Trends Anal. Chem..

[2] Melchert W.R., Reis B.F., Rocha F.R.P. (2012). Green chemistry and the evolution of flow analysis. A review. Anal. Chim. Acta.

[3] Miró M., Hansen E.H. (2013). On-line sample processing involving microextraction techniques as a front-end to atomic spectrometric detection for trace metal assays: A review. Anal. Chim. Acta.

[4] Trojanowicz M., Kołacińska K. (2016). Recent advances in flow injection analysis. Analyst.

[5] O’Connor Šraj L., Almeida M.I.G.S., Swearer S.E., Kolev S.D., McKelvie I.D. (2014). Analytical challenges and advantages of using flow-based methodologies for ammonia determination in estuarine and marine waters. Trends Anal. Chem..

[6] Genfa Z., Dasgupta P.K. (1989). Fluorometric measurement of aqueous ammonium ion in a flow injection system. Anal. Chem..

[7] Kerouel R., Aminot A. (1997). Fluorometric determination of ammonia in sea and estuarine waters by direct segmented flow analysis. Mar. Chem..

[8] Lin K., Zhu Y., Zhang Y., Lin H. (2019). Determination of ammonia nitrogen in natural waters: Recent advances and applications. Trends Environ. Anal. Chem..

[9] Kolev S.D., Fernandes P.R.L.V., Satinsky D., Solich P. (2009). Highly sensitive gas-diffusion sequential injection analysis based on flow manipulation. Talanta.

[10] Segundo R.A., Mesquita R.B.R., Ferreira M.T.S.O.B., Teixeira C.F.C.P., Bordalo A.A., Rangel A.O.S.S. (2011). Development of a sequential injection gas diffusion system for the determination of ammonium in transitional and coastal waters. Anal. Methods.

[11] Timofeeva I.I., Bulatov A.V., Moskvin A.L., Kolev S.D. (2015). A gas-diffusion flow injection method coupled with online solid–liquid extraction for the determination of ammonium in solid samples. Talanta.

[12] Wang L., Cardwell T.J., Cattrall R.W., Luque de Castro M.D., Kolev S.D. (2000). Pervaporation-flow injection determination of ammonia in the presence of surfactants. Anal. Chim. Acta.

[13] Wang L., Cardwell T.J., Cattrall R.W., Luque de Castro M.D., Kolev S.D. (2003). Determination of ammonia in beers by pervaporation flow injection analysis and spectrophotometric detection. Talanta.

[14] Mornane P., van den Haaka J., Cardwell T.J., Cattrall R.W., Dasgupta P.K., Kolev S.D. (2007). Thin layer distillation for matrix isolation in flow analysis. Talanta.

[15] Almeida M.I.G.S., Estela J.M., Segundo M.A., Cerdà V. (2011). A membraneless gas-diffusion unit – multisyringe flow injection spectrophotometric method for ammonium determination in untreated environmental samples. Talanta.

[16] Alahmad W., Pluangklang T., Mantim T., Cerdà V., Wilairat P., Ratanawimarnwong N., Nacapricha D. (2018). Development of flow systems incorporating membraneless vaporization units and flow-through contactless conductivity detector for determination of dissolved ammonium and sulfide in canal water. Talanta.

[17] Sramkova I., Horstkotte B., Sklenarova H., Solich P., Kolev S.D. (2016). A novel approach to lab-in-syringe head-space single-drop microextraction and on-drop sensing of ammonia. Anal. Chim. Acta.

[18] Timofeeva I., Khubaibullin I., Kamencev M., Moskvin A., Bulatov A. (2015). Automated procedure for determination of ammonia in concrete with headspace single-drop micro-extraction by stepwise injection spectrophotometric analysis. Talanta.

[19] Luque de Castro M.D., Gamiz-Gracia L. (2000). Analytical pervaporation: An advantageous alternative to headspace and purge-and-trap techniques. Chromatographia.

[20] Giakisikli G., Anthemidis A.N. (2018). Automatic pressure-assisted dual-headspace gas-liquid microextraction. Lab-in-syringe platform for membraneless gas separation of ammonia coupled with fluorimetric sequential injection analysis. Anal. Chim. Acta.

[21] Martínez E., Lacorte S., Llobeta I., Viana P., Barceló D. (2002). Multicomponent analysis of volatile organic compounds in water by automated purge and trap coupled to gas chromatography–mass spectrometry. J. Chromatogr. A.

[22] Zhu Y., Chen J., Yuan D., Yang Z., Shi X., Li H., Jin H., Ran L. (2019). Development of analytical methods for ammonium determination in seawater over the last two decades. Trends Anal. Chem..

[23] Wang P.-Y., Wu J.-Y., Chen H.-J., Lin T.-Y., Wu C.-H. (2008). Purge-and-trap ion chromatography for the determination of trace ammonium ion in high-salinity water samples. J. Chromatogr. A.

[24] Zhu Y., Yuan D., Lin H., Zhou T. (2016). Determination of ammonium in seawater by purge-and-trap and flow injection with fluorescence detection. Anal. Lett..

[25] Lin T.-Y., Pan Y.-T., Lee H.-Y., Wang P.-Y., Wu C.-H. (2012). Markedly enhanced purge-and-trap performance and efficiency for the determination of ammonium ion in high-salinity water samples. J. Chin. Chem. Soc..

[26] Sander R. (2015). Compilation of Henry’s law constants (version 4.0) for water as solvent. Atmos. Chem. Phys..

[27] Saridara C., Brukh R., Mitra S. (2006). Development of continuous on-line purge and trap analysis. J. Sep. Sci..

[28] Inczedy J., Lengyel T., Ure A.M. (1998). International Union of Pure and Applied Chemistry, Compendium of Analytical Nomenclature (definitive rules 1997).

[29] Marques K.L., Pires C.K., Santos J.L.M., Zagatto E.A.G., Lima J.L.F.C. (2007). A multi-pumping flow system for chemiluminescent determination of ammonium in natural waters. Int. J. Environ. Anal. Chem..

[30] Oliveira S.M., Lopes T.I.M.S., Tóth I.V., Rangel A.O.S.S. (2007). A multi-commuted flow injection system with a multi-channel propulsion unit placed before detection: Spectrophotometric determination of ammonium. Anal. Chim. Acta.

[31] Hong L., Sun X., Wang L. (2009). Determination of ammonia in water using flow injection analysis with automatic pervaporation enrichment. Anal. Lett..

[32] Federation, Water Environmental, and American Public Health Association (1999). Standard Methods for the Examination of Water and Wastewater.

[33] Giakisikli G., Trikas E., Petala M., Karapantsios T., Zachariadis G., Anthemidis A. (2017). An integrated sequential injection analysis system for ammonium determination in recycled hygiene and potable water samples for future use in manned space missions. Microchem. J..

