# Supplementary material for: Automatic On-Line Purge-and-Trap Sequential Injection Analysis for Trace Ammonium Determination in Untreated Estuarine and Seawater Samples
Source: Molecules. 2020 Mar 29;25(7):1569. doi: 10.3390/molecules25071569 (PMC7180869; doi:10.3390/molecules25071569)
Supplement: Supplementary file 1 [file molecules-25-01569-s001.pdf]

Supplementary Materials

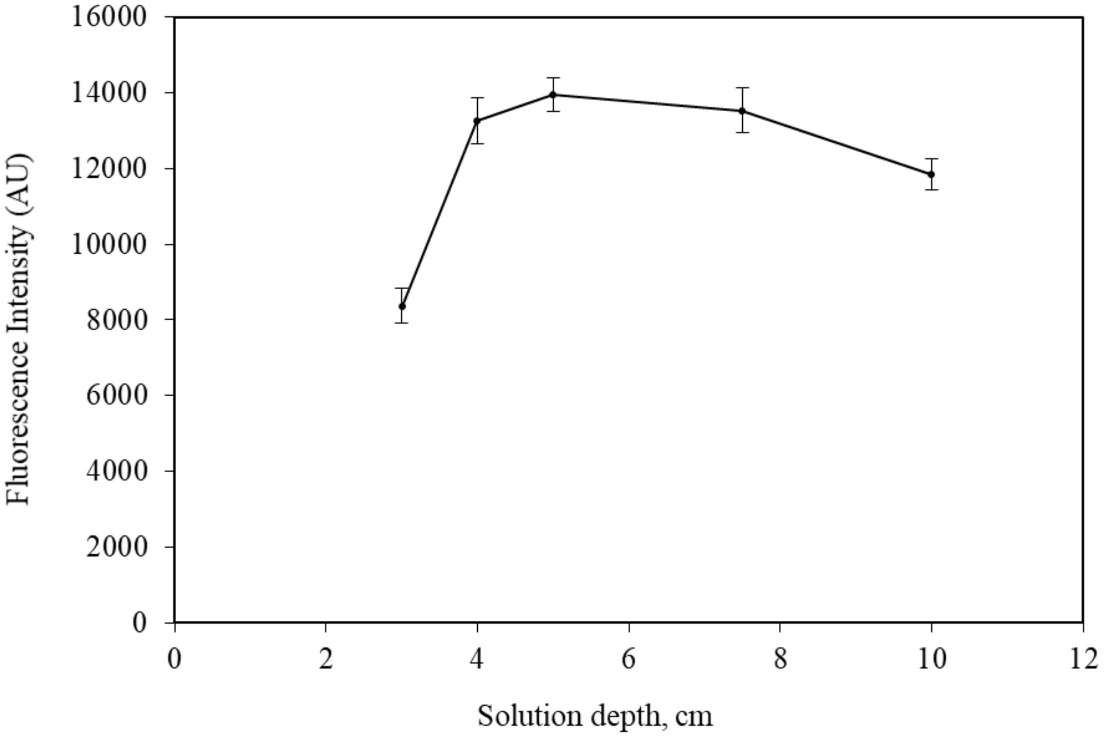

Figure S1. Effect of solution depth in purge-vessel on intensity of 5.0 μg NH_4^+. Error bars were calculated based on standard deviation (± 1s). NaOH volume = 1000 μL (1.0 mol L^{-1} NaOH); Trapping solution: 300 μL, 0.001 mol L^{-1} HCl; Purge-gas flow rate = 75 mL min^{-1}.

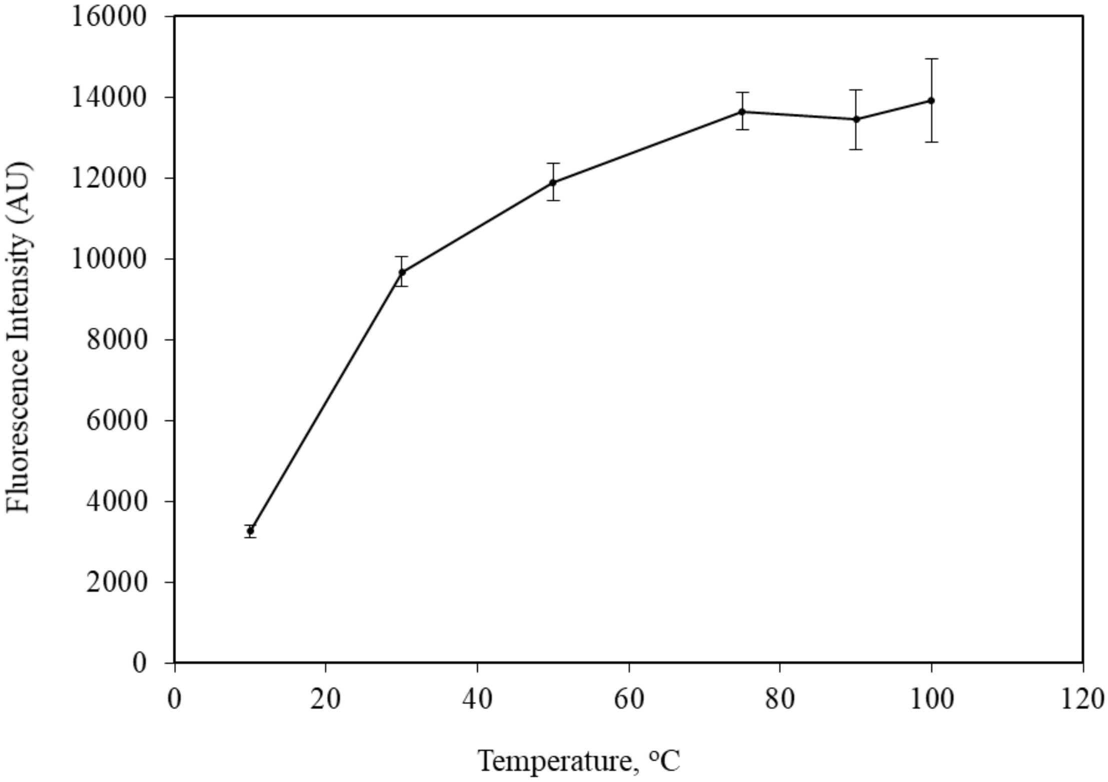

Figure S2. Effect of purge-gas flow rate of the on the sensitivity of the method. Error bars were calculated based on standard deviation (± 1s). Other parameters as in Fig. S1. .

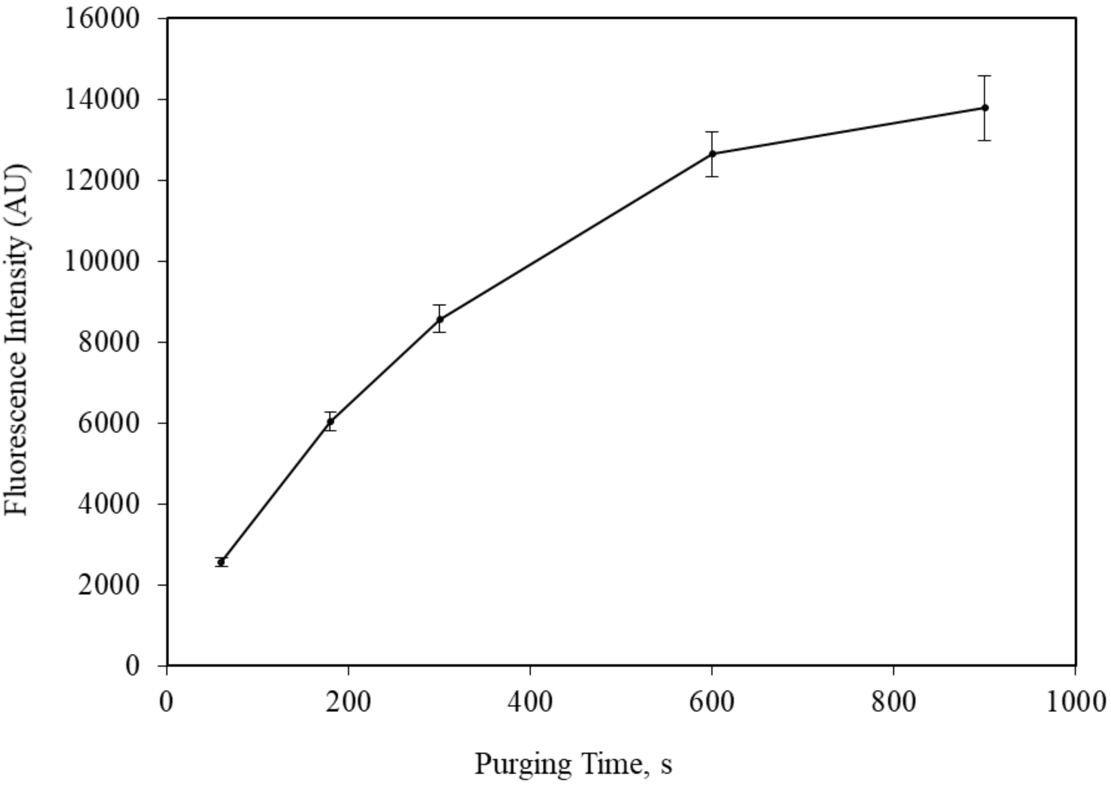

Figure S3. Effect of purging time on the fluorescence intensity of 50.0 μg L^{-1} NH_4^+. Error bars were calculated based on standard deviation (± 1s). All other parameters as in Fig. S1..
